# Infant hydrocephalus: what valve first?

**DOI:** 10.1007/s00381-021-05326-1

**Published:** 2021-08-17

**Authors:** Benjamin J. Hall, Conor S. Gillespie, Geraint J. Sunderland, Elizabeth J. Conroy, Dawn Hennigan, Michael D. Jenkinson, Benedetta Pettorini, Conor Mallucci

**Affiliations:** 1 Department of Neurosurgery, Alder Hey Children’s NHS Trust, Liverpool, UK; 2 Aintree University Hospitals NHS Foundation Trust, Liverpool, UK; 3 Institute of Systems, Molecular and Integrative Biology, University of Liverpool, Biosciences Building, Crown Street, Liverpool, L69 7BE UK; 4 Institute of Infection Veterinary and Ecological Sciences, University of Liverpool, Liverpool, UK; 5 Liverpool Clinical Trials Centre, University of Liverpool, Liverpool, UK

**Keywords:** Hydrocephalus, Valve, Shunt, Selection, Choice, Infant

## Abstract

**Purpose:**

To review the use of different valve types in infants with hydrocephalus, in doing so, determining whether an optimal valve choice exists for this patient cohort.

**Methods:**

We conducted (1) a literature review for all studies describing valve types used (programmable vs. non-programmable, valve size, pressure) in infants (≤ 2 years) with hydrocephalus, (2) a review of data from the pivotal BASICS trial for infant patients and (3) a separate, institutional cohort study from Alder Hey Children’s Hospital NHS Foundation Trust. The primary outcome was any revision not due to infection.

**Results:**

The search identified 19 studies that were included in the review. Most did not identify a superior valve choice between programmable and non-programmable, small compared to ultra-small, and differential pressure compared to flow-regulating valves. Five studies investigated a single-valve type without a comparator group. The BASICS data identified 391 infants, with no statistically significant difference between gravitational and programmable subgroups. The institutional data from our tertiary referral centre did not reveal any significant difference in failure rate between valve subtypes.

**Conclusion:**

Our review highlights the challenges of valve selection in infant hydrocephalus, reiterating that the concept of an optimal valve choice in this group remains a controversial one. While the infant-hydrocephalic population is at high risk of valve failure, heterogeneity and a lack of direct comparison between valves in the literature limit our ability to draw meaningful conclusions. Data that does exist suggests at present that there is no difference in non-infective failure rate are increasing in number, with the British valve subtypes in infant hydrocephalus, supported by both the randomised trial and institutional data in this study.

**Supplementary information:**

The online version contains supplementary material available at 10.1007/s00381-021-05326-1.

## Introduction

Ventriculoperitoneal (VP) shunts are the mainstay of treatment for hydrocephalus, especially in neonates and infants. Infant hydrocephalus incidence is between 1 and 32 per 10,000 births. Aetiology varies and often impacts clinical outcome [1]. Shunt failure and subsequent revisions constitute a significant part of the paediatric neurosurgeon’s workload, resulting in both financial cost [2, 3] and repeat patient exposure to the risks associated with surgery [4]. Reducing the likelihood of shunt failure is therefore crucial, and a number of influential variables have been identified.

Younger age is a significant determinant of shunt failure, with neonates and infants experiencing significantly higher rates of shunt complications, shunt failure and therefore revisions, compared to older children [4]. Within the infant population, it is therefore critical to optimise all other independent variables to mitigate risk of failure.

Randomised trials examining various aspects of shunt systems are increasing in number, with the British Antibiotic and Silver Impregnated Catheters for ventriculoperitoneal Shunts (BASICS) multi-centre randomised controlled trial [5] highlighting the superiority of antibiotic-impregnated shunts and, more recently, the CSF Shunt Entry Site Trial (ENTRY): examining the role of catheter position in determining shunt survival [6]. Kestle et al. reviewed the number of prospective multi-centre studies in paediatric hydrocephalus and identified only two that assessed the efficacy of different kinds of valves and their performance, leading to the conclusion that ‘valves are all the same’ [7]. However, since these trials [8, 9] were published in 1998 and 1999, the valve market has grown exponentially. Fixed, programmable, anti-siphon devices, flow regulation: for every obstacle that accompanies CSF diversion, a valve mechanism has been designed to tackle it. This breadth of variety has, however, resulted in very little rigorous comparison at a randomised control level, particularly in infant populations.

The adage established in the 1990s that ‘valves are all the same’ has arguably been replaced with ‘stick with what works’, with surgeon preference and experience now featuring heavily in system selection [10]. De novo shunt insertions typically have some of the most variable outcomes in failure rates and valve survival. This is largely due to the number of factors that are known to impact time to shunt failure: aetiology, age, infection, surgeon experience and shunt system all contribute to the likelihood of success [4].

While failure rate is typically the primary outcome in shunt comparison, it remains to be seen whether it is the only useful outcome measure. The cognitive impact of hydrocephalus is well recognised, particularly in the infant population; however, very little comparison has been performed between long-term follow-up of patient cognition in different valve groups. Alternative outcome measures, such as ventricular size and head circumference, are also under-investigated [11, 12].

Given that many de novo shunts are inserted in the infant population, ensuring the optimal valve is selected at this point of insertion is critical in minimising failure rates. This study seeks to describe the trends in de novo valve selection for the infant hydrocephalus population, as well as ascertain whether any single-valve system is superior to others in this population.

## Methods

We conducted three separate analyses as part of this review. Firstly, we carried out a literature review for all studies describing valve types used (programmable vs. non-programmable, valve size, pressure) in infants (≤ 2 years) with hydrocephalus, including both comparative and non-comparative studies. Second, we carried out a post hoc analysis of data from the pivotal BASICS trial for infant patients only. Third, we examined a separate, institutional cohort from Alder Hey Children’s Hospital NHS Foundation Trust of all valves used for infant hydrocephalus patients from February 2010 to September 2018.

## Literature review

A literature search was conducted of the PubMed registry on the 12th of February 2021, of all articles published between 1995 and 2020, with the search terms ‘hydrocephalus’, ‘infant’, ‘neonate’ and ‘valve’. Suitable articles for inclusion were those that included manuscripts that reviewed or assessed the efficacy of VP shunts in neonates and infants, including both comparative and non-comparative studies of valve types. All articles were screened by two independent reviewers (BJH and CSG). If disagreements occurred over selection, senior authors were selected for clarification (BP and CM). Randomised and non-randomised studies were included. If articles reported mixed data sets containing results of both infants and children under the age of 18, studies with a median age of < 2 years old or with at least an 80% majority infant cohort were included.

## Data collection

Retrospective analysis of derived data sets from the BASICS trial, full details of methodology for which are as previously published [5]. Relevant data sets were collated from prospectively generated databases on the basis of anonymised unique patient trial IDs.

## Study participants

One thousand six hundred five patients receiving de novo VP shunt insertion were recruited to the BASICS trial between June 2013 and October 2017 and randomised to receive either plain silicon, silver-impregnated or antibiotic-impregnated shunt catheters. Patients of any age and of all hydrocephalus aetiologies were eligible for inclusion. From this data set, 391 patients ≤ 2 years are included in the analysis presented.

## Valve grouping

For pragmatic analysis, the following schemes were devised to classify the wide variety of valves implanted in the BASICS trial. Valves with an integrated gravitational unit (programmable and non-programmable) produced by Miethke were classified as gravitational, and all other valve types were classified as non-gravitational. In addition, valves were dichotomised as being either programmable or non-programmable on the basis of whether they have a feature to adjust the valve opening pressure. Combining these 2 schemes resulted in 4 valve combinations: non-gravitational non-programmable (NGNP), non-gravitational programmable (NGP), gravitational non-programmable (GNP) and gravitational programmable (GP).

## Outcomes

Participants in the BASICS trial were followed up to a maximum of 24 months (median 22 months). Shunt revision for any cause and reason for revision were secondary endpoints. The outcome of interest in this analysis was valve revision rate. This was defined as any revision not due to infection as recorded within the BASICS final analysis. This definition encompasses all non-infective failures including, but not limited to, mechanical obstruction and functional failure (both under and over-drainage). Due to a focus on valve design, failures due to infection were excluded from formal statistical analysis.

## Statistical analysis

Data management and statistical analysis were performed in R Studio with R version 4.02.

Time to revision is illustrated using Kaplan–Meier curves. Comparison of dichotomous variables is presented descriptively and compared with a chi-square test. Time to revision analysis is illustrated using Kaplan–Meier survival curves based on Cox proportional hazards models adjusting for covariates of interest. Statistical significance threshold was set at *p* < 0.05. Data was assumed to be missing at random and therefore no imputation was carried out. No adjustment for multiplicity has been performed, rather, inferences are drawn from the statistical significance of the results reported.

## Alder Hey institutional data

Clinical notes of all patients receiving de novo Miethke fixed pressure (paediGAV) valves at Alder Hey Children’s Hospital NHS Foundation Trust between February 2010 and September 2018 were collected. Miethke proGAV®2.0 programmable valves were not used in the Trust until a later date, therefore notes of all patients receiving de novo proGAV2.0® valves from January 2014 to September 2018 were also collected. Data was collected according to the following headings: (i) patient demographic, (ii) symptomatology (pre and postoperatively), (iii) indication for shunt/valve insertion, (iv) frequency of surgical intervention, (v) frequency of valve setting alteration and (vi) ventricular linear metrics. Over-drainage was defined according to a combination of ventricular morphology, symptomatic presentation of the patient and, if performed, ICP monitor values. The rationale for selecting appropriate replacement valves in the cases where revision was necessary was decided on a case by case basis. Gravitational unit for proGAV2.0® choice was made according to Miethke guidance: 20-cm H_2_O for those under 5 years of age and 25-cm H_2_O for over 5 years. Any cases in which available data regarding shunt pressures at the time of insertion was not available were excluded.

Patients > 2 years of age at the time of initial shunt insertion were excluded from further analysis. Shunt system failure was defined as ‘requiring revision or surgical re-intervention not due to infection’. A subgroup of patients was also analysed, wherein those experiencing failure within 3 months of initial insertion were excluded, accounting for immediate surgical or inpatient complications as confounding factors in failure rate. Only shunt revisions secondary to mechanical failure were included; those resulting from infection, CSF leak or catheter failure were excluded.

For all programmable valve patients, preoperative and postoperative imaging was assessed to determine changes in ventricular linear metrics. T2-weighted MRI scans were used to measure frontal and occipital horn ratio (FOHR) and frontal and occipital horn width ratio (FOHWR). In the absence of T2-weighted MRI, CT scans were used. All measurements were taken at the level of the intraventricular foramen of Monro, as identified on axial images. The FOHR was defined as the mean value of the frontal and occipital horn width divided by twice the widest biparietal diameter (BPD) [13, 14]. FOHWR was then defined as the average of the maximum width of the individual frontal and occipital horns, divided by twice the widest BPD [15]. The closest available scans prior to and following shunt insertion/revision were used to assess any changes. Ventricular metric data was not available for non-programmable valves used at this centre.

The latest versions of Microsoft Excel and SPSS statistical software were used for data collection and statistical analysis, respectively.

## Results

### Literature review

The search terms returned 173 results, with 19 articles included after title and abstract screening (Supplementary Table 1). Three randomised control trials comparing valve types were identified [8, 9, 11]. All other studies were multi-centre prospective, or single-centre prospective or retrospective studies. Ten included infants specifically (less than 2 years old), and seven studies included infants alongside older children. In almost all of these studies, infants constituted greater than 80% of the study cohort [11, 12], or the median age was under 1 year of age [8, 16–19].

### Valve comparison in the literature

#### Programmable vs. fixed valve (including gravitational units) comparison

Investigation of the efficacy and safety of programmable valves was the most prevalent aim of the studies identified. Five studies examined only a single model of programmable valve, without comparing to any other mechanisms or manufacturers; the conclusions across the articles being that programmable valves are indeed safe and efficacious. Another study compared two programmable valves, this time from the same manufacturer (Sophy/Polaris), finding them equally efficacious and without any significant discrepancies [20].

One study of only infants identified no difference between shunt survival for adjustable (Miethke proGAV) compared to fixed valves (Orbis-Sigma) (*p* = 0.18) but reported that the adjustable valve cohort was more likely to suffer from over-drainage [21]. Another study of 50 infants failed to identify a difference in shunt survival between differential pressure compared to flow-regulating valves (*p* = 0.72) but conversely identified a lower incidence of over-drainage in flow-regulating valves [22]. There was no difference in failure rates between programmable and pressure-controlled valves in a large study of 253 patients (*p* = 0.11) [17]; however, this was a mixed paediatric cohort, with the median age at shunt insertion being 30 days old. Another large study of a mixed paediatric population did not identify a significant difference in shunt failure rates of programmable and fixed shunts (hazard ratio (HR) 0.8 (programmable), 95% CI 0.6–1.1). In this study, the mean age at insertion was 131 weeks, with 56% of the study population under 6 months of age [16]. A further multi-centre retrospective study of only neonates did not identify a difference in shunt complications among fixed pressure, anti-siphon, flow control and adjustable valves (no *p*-value stated) [23].

Several studies did compare programmable and non-programmable valves, including the two premillennial RCTs that both confirmed that there is no significant difference in either safety or efficacy between these valve types. More recently, Riva-Cambrin et al. compared non-programmable and programmable valves according to mechanism but also by manufacturer, once again confirming the notion that neither is superior with similar failure rates.

#### Low-pressure vs. medium/high-pressure valve comparison

A randomised control trial of 40 patients with mainly infant hydrocephalus patients identified no difference in complications and incidence of redo shunt surgery in the low- and medium-pressure groups (*p* = 0.5614) [11]. Although hypothesised that low-pressure valves facilitate more CSF drainage, with increased predisposition to proximal occlusion, this was not supported by the study. While Robinson et al. identified more complications and higher revision rates in low-pressure valves [12], Breimer et al. found that low-pressure valves were a safe choice in infants [24].

#### Differential pressure or fixed/program vs. flow-regulating valve comparison

Several studies considered the use of flow-regulating valves in the infant population. These were compared to fixed differential pressure valves [22] and programmable valves [21]; though neither study identified a survival difference, both did identify reduced rates of over-drainage with the use of the flow-regulating valves. Beuariat et al., on the other hand, did identify a significant difference in shunt survival, with differential pressure valves having significantly poorer survival than the flow-regulating valves (HR 1.77, 95% CI 1.19–2.64) [19].

#### Size of valve (ultra-small vs. standard) comparison

Valve size has previously been linked to performance and was noted in this review. Kahilogullari et al. identified that early complication rates were lower in ultra-small valves but did not identify any difference according to valve mechanism [23]. Reed et al. did not, however, find any difference in comparing ultra-small to standard valves, with similar 12-month survival rates [25].

### BASICS trial data

Four hundred forty-six patients ≤ 2 years of age were identified in the BASICS database who had undergone de novo VP shunt insertions during the trial period. Thirteen patients had missing data relating to valve type or follow-up and were therefore excluded from further study, thus a total population of 433 patients was available. The overall proportion of valve types inserted was as follows: *n* = 256 (59.1%) non-gravitational and *n* = 177 (40.9%) gravitational, *n* = 374 (86.4%) non-programmable and *n* = 59 (13.6%) programmable. Within these, 228 (52.7%) valves were NGNP, 28 (6.5%) were NGP, 146 (33.7%) were GNP and 31 (7.2%) were GP.

One hundred seventy of 433 (39.3%) patients required revision of VP shunt for any reason within 2 years. Forty-two (9.7%) failures were due to infection and are excluded from the formal statistical analyses presented in Table 1.

**Table 1:** Number and proportion of non-infected VP shunt revisions by valve feature (gravitational/non-gravitational) and valve subtype

Valve type	Total *n* (%)	Mechanical failure failure *n* (%)	X^2^	*p *value
All valves	391	128 (32.7)		
Valve feature				
Non-gravitational	236 (60.4)	72 (30.5)	1.099	0.294
Gravitational	155 (39.6)	56 (36.1)		
Non-programmable	337 (86.2)	112 (33.2)	0.135	0.713
Programmable	54 (13.8)	16 (29.6)		
Valve subtype				
NGNP	211 (54.0)	65 (30.8)	1.843	0.606
NGP	25 (6.4)	7 (28.0)		
GNP	126 (32.2)	47 (37.3)		
GP	29 (7.4)	9 (31.0)		

**Fig. 1:**
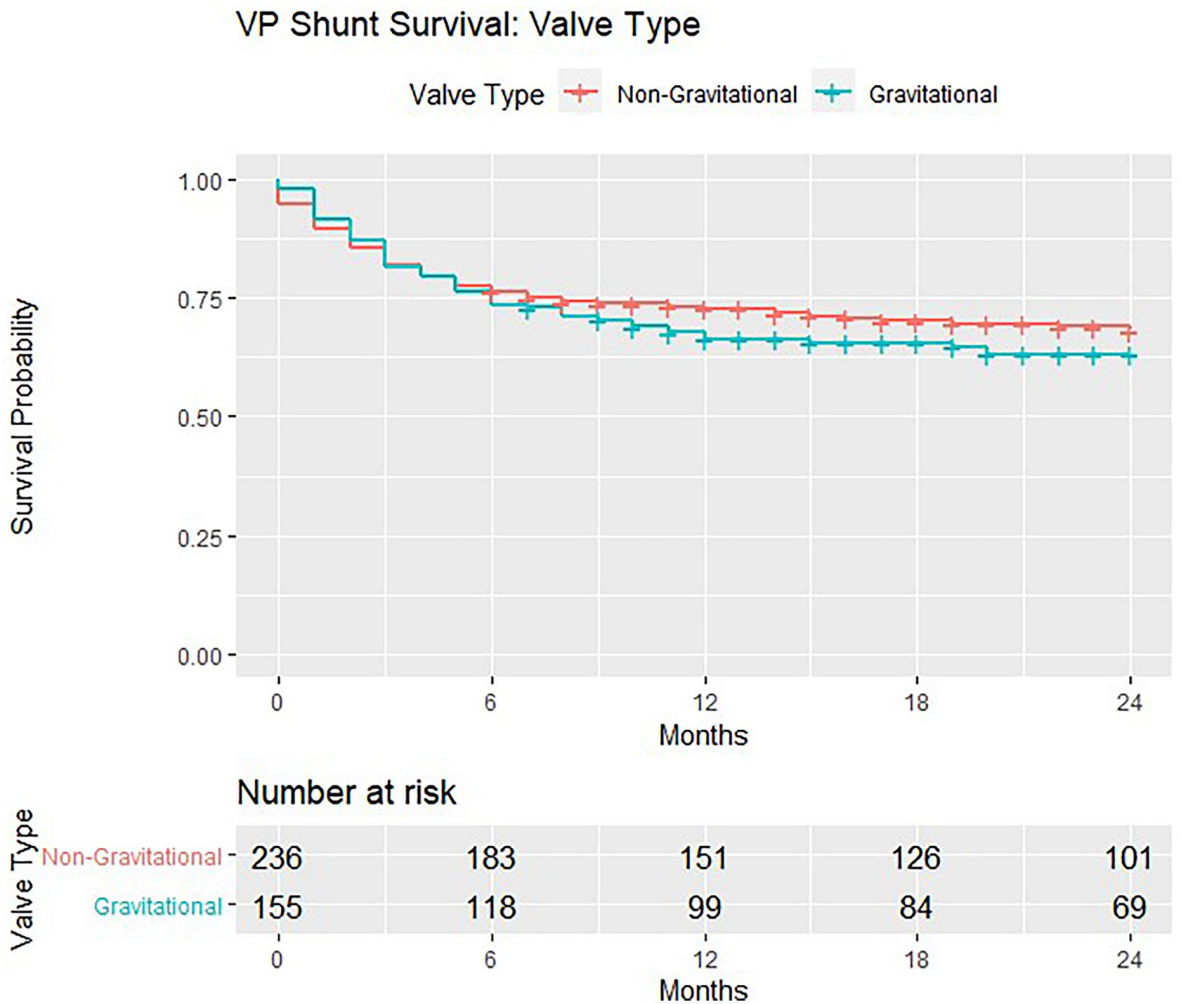
Kaplan–Meier survival estimates for non-gravitational vs. gravitational valves implanted in the BASICS trial

**Fig. 2:**
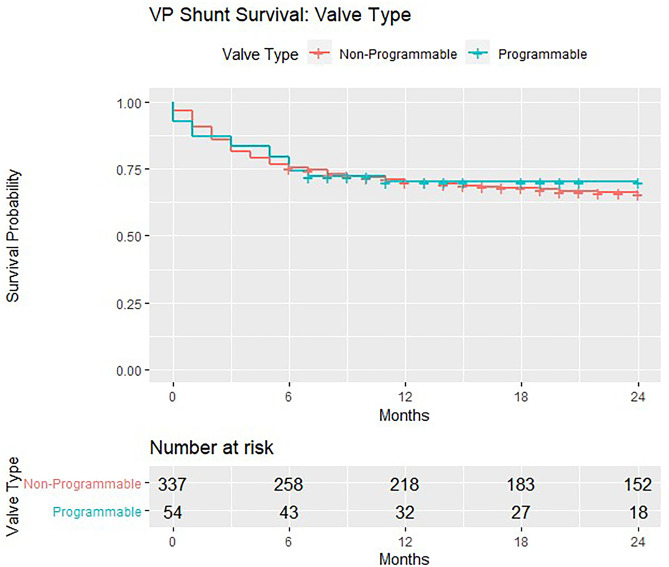
Kaplan–Meier survival estimates for non-programmable vs. programmable valves implanted in the BASICS trial

**Fig. 3:**
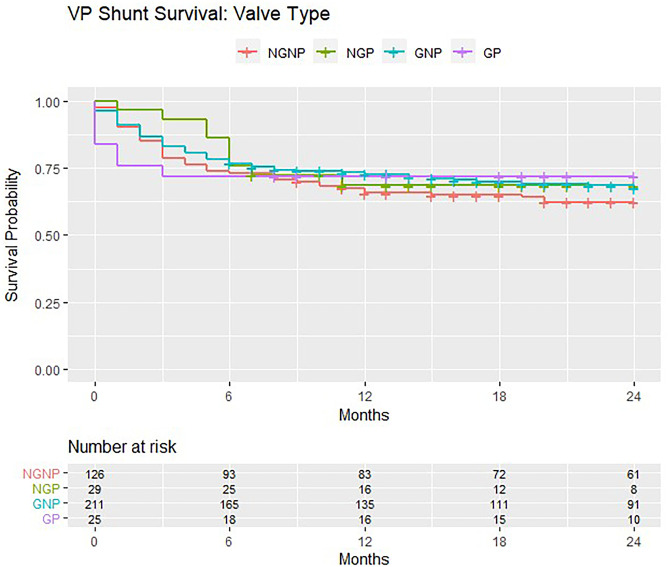
Kaplan–Meier survival estimates for the various valve types (NGNP/NGP/GNP/GP) implanted in the BASICS trial

In the 391 patients eligible for formal analyses, there were more mechanical failures in gravitational valves (*n* = 56/155, 36.1%) compared with non-gravitational models (*n* = 72/236, 30.5%). There were more mechanical failures in non-programmable valves (*n* = 112/337, 33.2%) compared with programmable models (*n* = 16/54, 29.6%). None of these differences achieved significance on chi-square test for independence (Table 1). VP shunt survival trends are demonstrated in Kaplan–Meier curves (Figs. 1, 2 and 3). The survival curves for covariates are seen to cross suggesting non-proportional hazards, precluding further calculation of hazard ratios and tests for significance.

### Institutional alder hey data

Two hundred eight patients under the age of 2 at initial shunt insertion were identified, of which 7 valves were unidentifiable. Of the remaining valves, 28 went on to have revision secondary to infection, 27 were due to catheter failure, 1 due to distal disconnection, 2 due to CSF leaks, 1 due to CSF resorption failure and 1 cause unknown. Thus, 141 patients were included for analysis.

All valves analysed in this cohort, both programmable and non-programmable, had gravitational components and were therefore either NPG or PG. One hundred thirteen of 141 (80.1%) valves were non-programmable and 28 (19.9%) were programmable. All non-programmable valves were Miethke paediGAV® fixed differential pressure valves of varying pressures: *n* = 12 (8.5%) 4/24, *n* = 7 (5.0%) 9/24 and *n* = 94 (66.7%) 9/29. All programmable valves (*n* = 28) in this set were proGAV2.0® valves, with 20-cm H_2_O-gravitational units. Those receiving non-programmable valves were slightly younger (mean age 113 days) on insertion of their de novo valves compared to the programmable valve population (mean age 161 days), though this was not significant (*p* = 0.1).

**Fig. 4:**
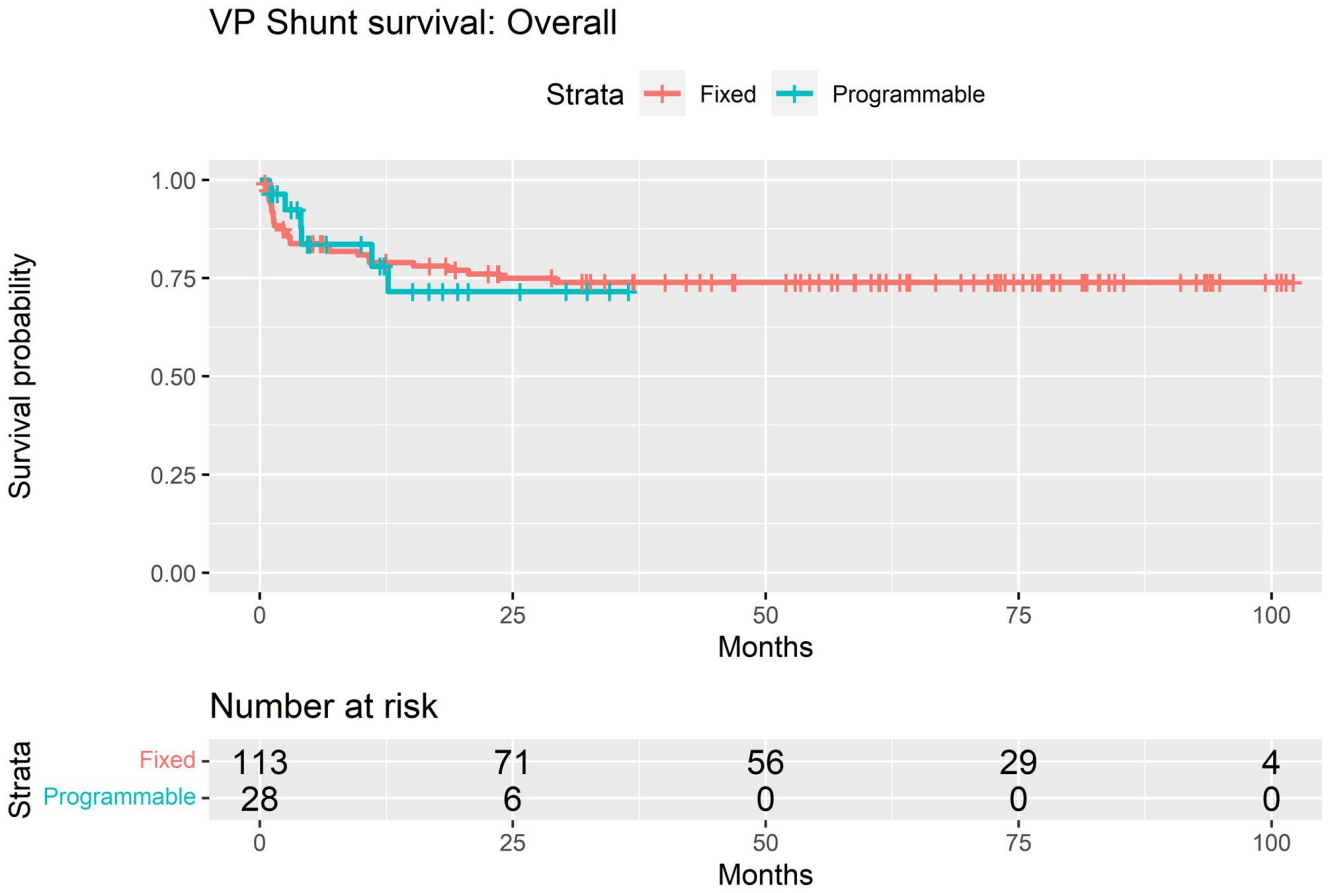
Kaplan–Meier survival estimates for non-programmable vs. programmable valves implanted in the Institutional Alder Hey cohort

Overall, 34 of 141 (24.1%) required revision of their de novo shunt system: 28 of 113 (24.7%) non-programmable and 6 of 28 programmable (21.4%). Two-year survival for non-programmable valves in the overall cohort was 75.0%, compared to 71.5% in programmable valves (*p* = 0.95) (Fig. 4). Eighteen of 141 (12.8%) required shunt revision within 3 months of insertion: 16 of 18 (88.9%) non-programmable valves and 2 of 18 (11.1%) programmable valves.

**Fig. 5:**
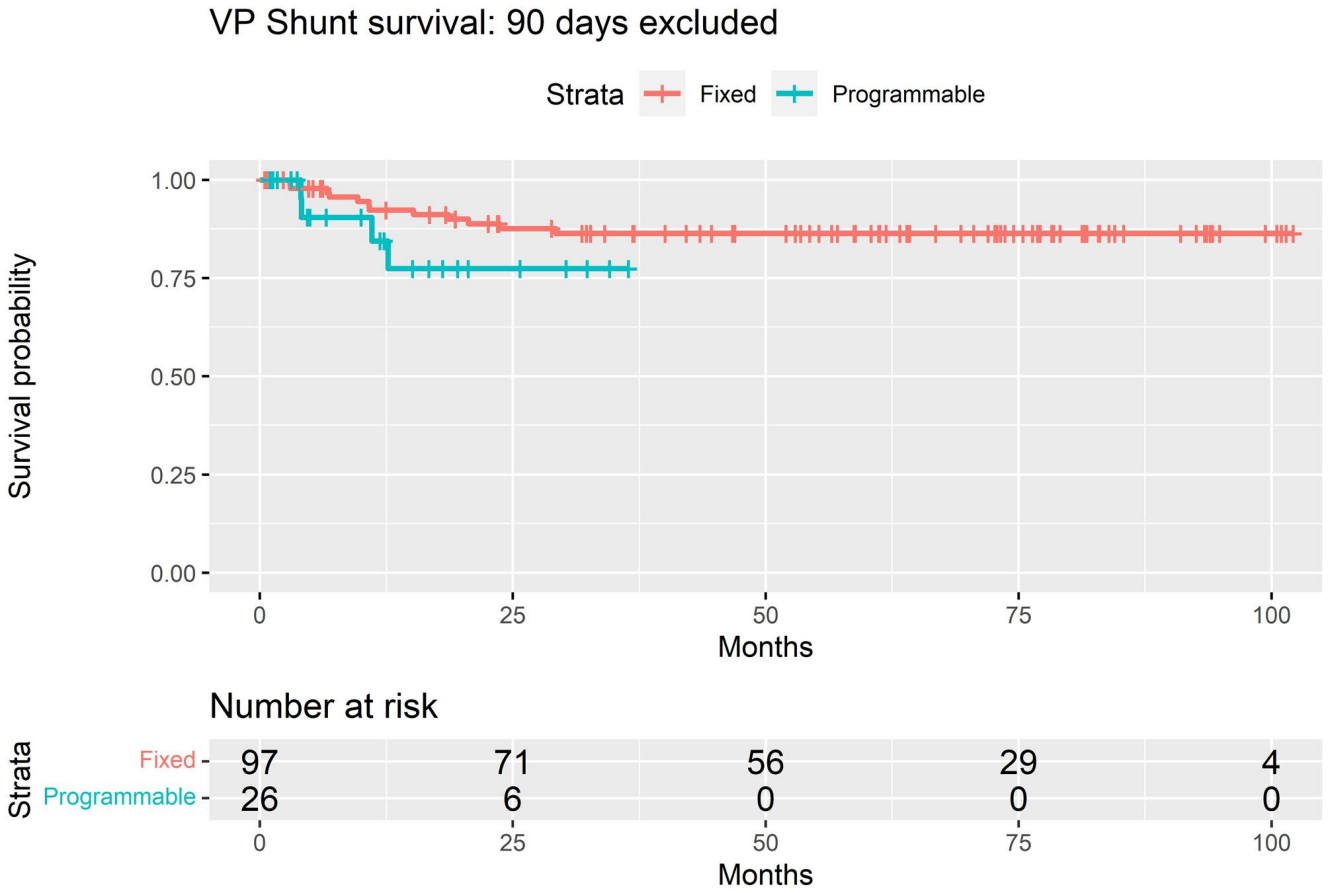
Kaplan–Meier survival estimates for non-programmable vs. programmable valves implanted in the Institutional Alder Hey cohort (with shunts surviving < 90 days excluded)

Of the 123 patients whose shunts survived beyond 3 months, the valve subgroups were as follows: *n* = 10 (8.1%) 4/24, *n* = 6 (4.9%) 9/24, *n* = 81 (65.9%) 9/29 and *n* = 26 (21.1%) proGAV2.0®. Sixteen of the 123 (13.0%) patients that had shunt systems survive beyond 3 months eventually required revision, 12 of 97 (12.4%) non-programmable and 4 of 26 (15.4%) programmable. Two-year survival rate when excluding those that failed within 3 months of insertion was 87.6% for non-programmable valves and 77.4% in programmable valves (*p* = 0.2) (Fig. 5).

Across the entire cohort of valve insertions, a significant decrease was seen in both mean FOHR and FOHWR. The mean decrease in FOHR was 0.014 (95% CI 0.006–0.023, *p* = 0.002), while the mean decrease in FOHWR was 0.037 (95% CI 0.005–0.069, *p* = 0.024).

## Discussion

Ventriculoperitoneal shunting and the use of differential pressure valves to facilitate this remain the backbone of modern CSF diversion. While there has been an exponential growth in the availability and variability of valve designs, there has been very little in the way of thorough comparison between them. Hydrocephalus is a heterogenous clinical entity, and the most complex challenges are often encountered in some of the youngest patients. The literature review here identified a number of papers that aim to compare valves according to mechanism, focusing particularly on infant cohorts.

The literature is predominantly composed of single-centre validation of single-type valves, all demonstrating similar failure and complication rates. The majority of studies could not identify a difference in shunt failure or complication rates for each valve type, but results may be hidden by reduced study power. One of the earliest RCTs to compare valve subtypes did not demonstrate a difference in failure rates between standard differential valves and externally programmable valves [8]. A systematic review that included non-programmable and programmable valves almost two decades later concluded that a superior valve choice in paediatric patients could still not be identified [26]. While many studies use mechanical failure as their primary outcome measure, it remains to be seen whether this is the most useful metric. Cognitive outcomes and quality of life have also been used, notably by the DRIFT trial in intraventricular haemorrhage and, more recently, by the International Infant Hydrocephalus Study (IIHS) [27]. By using tools such as the Health Utilities Index (HUI) Mark 2 and Denver II Developmental Screening Tool (DDST), IIHS provides an important schematic for future study designs, enabling a more comprehensive approach when comparing valve subtypes. Moreover, establishing 3 and 5-year outcomes as their a priori goal provides robust long-term follow-up data and should be encouraged in future studies.

Some studies [11, 12, 27], IIHS included, report ventricular metrics as outcome measures for comparing interventions, though the relationship between ventricular metrics and cognitive outcomes is unclear [28]. Evidence suggests that ventricular metrics are not necessarily associated with valve type or revision rate [11, 12], and while our data demonstrated an immediate reduction in ventricular size, we are unable to comment on the relevance of ventricular metrics at a later follow-up.

Unfortunately, time to first failure is especially challenging to investigate in a controlled manner due to the dynamic and multi-factorial processes that exist at de novo shunt insertion. Aetiology of hydrocephalus, infection and surgical experience are all confounding factors when it comes to orchestrating such a study [8].

Determining the current trends in chosen valve mechanism for the infant population is challenging (Supplementary Table 1), as many of these retrospective studies only comment on either one or a handful of valves, which may not necessarily reflect that centre’s typical practice. In both the BASICS dataset and our own data, we identified a predominantly non-programmable cohort (80.0–86.4%).

Programmable valves have been reported to have an 11.1% risk of mechanical failure rate, ranging from 10.0 to 59.0% in the literature for this cohort; in comparison to non-programmable which have been reported as close to zero previously [29]. Mechanical failure rates in programmable valves were similar between BASICS and our own data, at 29.6% and 24.0%, respectively.

The comparable survival rates between programmable and non-programmable valves across all data here raise the question as to why the former, often more costly, programmable valves should be opted for. This study focuses on de novo shunt systems, inserted into patients with newly diagnosed hydrocephalus, in which CSF over-drainage, for which programmable valves were designed, is not necessarily prevalent. Whether then this suggests better patient selection criteria for programmable valves is required, and that they may be better placed in the ‘valve revision’ setting in those where over-drainage is present, requires further investigation.

Our data is limited in its comparative ability given that all valves examined in our data set feature gravity-assist devices and therefore may not represent the community at large. While there was no significant difference (*p* = 0.95) in survival between valve subtypes, numerically there are notable differences between both curves comparing gravitational and non-gravitational valves. Whether these trends would eventually become of significance with greater follow-up warrants further investigation.

The multi-faceted nature of hydrocephalus makes rigorous investigation challenging, and the study lacks a number of insights which would also benefit from further study. While programmable valves are seen as a potential solution to CSF over-drainage, relatively little is still understood about the related ‘slit-ventricle’ syndrome and the impact of valve choice on this. Subtle changes to ventricular morphology are likely to have the greatest long-term impact in the infant population, and while many studies, our own included, investigate the efficacy of valves in managing acute hydrocephalus, their impact in preventing developmental and neurocognitive deficits is severely under-explored.

## Conclusion

Our data is comparable to the BASICS data and most of the literature in terms of reflecting the main choice in the UK for infant hydrocephalus is a fixed pressure valve. This attitude is presumably reflective of a lack of evidence for superiority of the more expensive programmable valves in preventing mechanical shunt failure. Despite the apparent wealth of data analysing various valve types, there is a lack of direct, objective comparison between them and the choice of appropriate outcome metrics. The paucity of rigorous comparison between valve subtypes in the infant population, as evidenced by the literature review performed in this study, is a failing on behalf of the global neurosurgical community and requires correction through the use of high-powered multi-centre trials. In view of the fact that the first shunt in such a vulnerable population is likely to be one of the most important decisions that a paediatric neurosurgeon would make, it is regrettable that a lack of evidence still pervades in aiding this decision.

In reality, new study methods need to be proposed: changing the emphasis from mechanical revision events as the only outcome metric. Future studies will need to be designed primarily studying outcome measures that would reflect likely cognitive outcome to help choose the right valve.

## Supplementary information

Below is the link to the electronic supplementary material.

Supplementary file1 (DOCX 20 KB)

## Data Availability

Anonymised data are available (on reasonable request) from the corresponding author.

## References

[1] Tully HM et al (2014) Infantile hydrocephalus: a review of epidemiology, classification, and causes. Eur J Med Genet. 10.1016/j.ejmg.2014.06.002 10.1016/j.ejmg.2014.06.002 PMC4334358 24932902

[2] Patwardhan RV et al (2005) Implanted Ventricular shunts in the United States: the billion-dollar-a-year cost of hydrocephalus treatment. Neurosurgery 56(1):139–44. 10.1227/01.neu.0000146206.40375.41 10.1227/01.neu.0000146206.40375.41 15617596

[3] Lim J et al (2018) The cost of hydrocephalus: a cost-effectiveness model for evaluating surgical techniques. J Paediatr Neurosurg 23(1):109–118. 10.3171/2018.6.PEDS17654 10.3171/2018.6.PEDS17654 30497214

[4] Merkler AE et al (2017) The rate of complications after ventriculoperitoneal shunt surgery. World Neurosurg. 10.1016/j.wneu.2016.10.136 10.1016/j.wneu.2016.10.136 PMC5326595 27826086

[5] Mallucci CL et al (2019) Antibiotic or silver versus standard ventriculoperitoneal shunts (BASICS): a multicentre, single-blinded, randomised trial and economic evaluation. The Lancet. 10.1016/S0140-6736(19)31603-4 10.1016/S0140-6736(19)31603-4 PMC6999649 31522843

[6] Whitehead et al (2021) The CSF Shunt Entry Site Trial (10/02/2021). Clinicaltrials.gov. Available from: https://www.clinicaltrials.gov/ct2/show/NCT02425761

[7] Kestle JR, Riva-Cambrin J (2019) Prospective multicenter studies in pediatric hydrocephalus. J Neurosurg: Pediatrics PED 23(2):135–141 10.3171/2018.10.PEDS18328 30717034

[8] Drake JM et al (1998) Randomized trial of cerebrospinal fluid shunt valve design in pediatric hydrocephalus. Neurosurgery 43(2):294–303; discussion 303 10.1097/00006123-199808000-00068 9696082

[9] Pollack IF et al (1999) A randomized, controlled study of a programmable shunt valve versus a conventional valve for patients with hydrocephalus. Hakim-Medos Investigator Group. 10.1097/00006123-199912000-00026 10.1097/00006123-199912000-00026 10598708

[10] Albright A (2010) Hydrocephalus shunt practice of experienced pediatric neurosurgeons. Child’s Nervous System. 10.1007/s00381-010-1082-5 10.1007/s00381-010-1082-5 20143074

[11] Sinha A et al (2012) Pediatric hydrocephalus: does the shunt device pressure selection affect the outcome? J Indian Assoc Pediatr Surg 17(2):54–57. 10.4103/0971-9261.93962 10.4103/0971-9261.93962 PMC3326822 22529548

[12] Robinson S et al (2002) Outcome analysis of initial neonatal shunts: does the valve make a difference? Paediatr Neurosurg 37(6):287–94. 10.1159/000066307 10.1159/000066307 12422042

[13] O’Hayon BB, Drake JM, Ossip MG, Tuli S (1998). Clarke M Frontal and occipital horn ratio: a linear estimate of ventricular size for multiple imaging modalities in pediatric hydrocephalus. Pediatr Neurosurg.

[14] Kulkarni AV, Measurement of ventricular size,  (1999). reliability of the frontal and occipital horn ratio compared to subjective assessment. Pediatr Neurosurg.

[15] Jamous M et al (2003) Frontal and occipital horn width ratio for the evaluation of small and asymmetrical ventricles. Pediatr Neurosurg 39:17–21. 10.1159/000070874 10.1159/000070874 12784072

[16] Riva-Cambrin J et al (2016) Risk factors for shunt malfunction in pediatric hydrocephalus: a multicenter prospective cohort study. J Neurosurg – Paediatr. 10.3171/2015.6.PEDS14670 10.3171/2015.6.PEDS14670 26636251

[17] Notarianni C et al (2009) Congenital hydrocephalus and ventriculoperitoneal shunts: influence of etiology and programmable shunts on revisions. J Neurosurg – Paediatr. 10.3171/2009.7.PEDS08371 10.3171/2009.7.PEDS08371 19951042

[18] Ahn ES et al (2007) The Strata programmable valve for shunt-dependent hydrocephalus: the pediatric experience at a single institution. Childs Nerv Syst 23:297–303. 10.1007/s00381-006-0236-y 10.1007/s00381-006-0236-y 17028879

[19] Beuriat PA et al (2017) Hydrocephalus treatment in children: long-term outcome in 975 consecutive patients. J Neurosurg – Paediatr. 10.3171/2017.2.PEDS16491 10.3171/2017.2.PEDS16491 28430083

[20] Martinez-Lage JF et al (2007) Management of neonatal hydrocephalus: feasibility of use and safety of two programmable (Sophy and Polaris) valves. Childs Nerv Syst. 10.1007/s00381-007-0512-5 10.1007/s00381-007-0512-5 17924120

[21] Henderson D et al (2020) A comparison between flow-regulated and adjustable valves used in hydrocephalus during infancy. Childs Nerv Syst. 10.1007/s00381-020-04552-3 10.1007/s00381-020-04552-3 32152667

[22] Jain H et al (2000) The treatment of infantile hydrocephalus: “differential-pressure” or “flow-control” valves. Childs Nerv Syst 16(4):242–6 10.1007/s003810050505 10855523

[23] Kahilogullari G et al (2018) Does shunt selection affect the rate of early shunt complications in neonatal myelomeningocele-associated hydrocephalus? A multi-center study. Turkish Neurosurg. 10.5137/1019-5149.JTN.18547-16.1 10.5137/1019-5149.JTN.18547-16.1 27593850

[24] Breimer GE et al (2012) Low-pressure valves in hydrocephalic children: a retrospective analysis. Childs Nerv Syst. 10.1007/s00381-011-1664-x 10.1007/s00381-011-1664-x PMC3284678 22205533

[25] Reed SW et al (2020) Neonatal hydrocephalus treatment with ultrasmall valve implantation. World Neurosurg. 10.1016/j.wneu.2019.09.043 10.1016/j.wneu.2019.09.043 31526889

[26] Baird NA et al (2014) Paediatric hydrocephalus; systematic literature review and evidence-based guidelines. J Neurosurg. 10.3171/2014.7.PEDS14321 10.3171/2014.8.PEDS14426 25988776

[27] Kulkarni AV et al (2018) International Infant Hydrocephalus Study (IIHS): 5-year health outcome results of a prospective, multicenter comparison of endoscopic third ventriculostomy (ETV) and shunt for infant hydrocephalus. Childs Nerv Syst 34(12):2391–2397. 10.1007/s00381-018-3896-5. Epub 2018 Jul 9 10.1007/s00381-018-3896-5 29987375

[28] Kulkarni AV, Donnelly R, Mabbott DJ, Widjaja E (2015) Relationship between ventricular size, white matter injury, and neurocognition in children with stable, treated hydrocephalus. J Neurosurg Pediatr 16:267–274. 10.3171/2015.1.PEDS14597 10.3171/2015.1.PEDS14597 26046689

[29] Mangano FT et al (2005) Early programmable valve malfunctions in pediatric hydrocephalus. J Neurosurg 103(6 Suppl):501–7. 10.3171/ped.2005.103.6.0501 10.3171/ped.2005.103.6.0501 16383248

