# Supplementary material for: Infant hydrocephalus: what valve first?
Source: Childs Nerv Syst. 2021 Aug 17;37(11):3485–95. doi: 10.1007/s00381-021-05326-1 (PMC8578053; doi:10.1007/s00381-021-05326-1)
Supplement: Supplementary file 1 — Supplementary file1 (DOCX 20 KB) [file 381_2021_5326_MOESM1_ESM.docx]

| **Supplementary table 1.** Results of literature search for valve selection choice in infant hydrocephalus patients. | | | | | | |
| --- | --- | --- | --- | --- | --- | --- |
| **Author** | **Study design** | **Objective** | **Cohort size**  **(details)** | **Follow-up** | **Main Results** | **Conclusions** |
| **Multicentre** | | | | | | |
| Drake et al, 1998 | Randomised control trial | Standard valve vs siphon control vs variable resistance valve | N=344  (median age of groups: 73, 78, 89 days) | Endpoint at 12 months; max. follow-up 36 months | Mechanical obstruction rate at 12 months:  Delta (anti-siphon): 33%  Standard (fixed): 34.2%  Orbis-Sigma (variable resistance): 27.0%  (p=0.24) | Type of valve does not affect shunt malfunction rates. |
| Pollack et al, 1999 | Randomised control trial | Codman-Hakim programmable valve vs conventional non-programmable of surgeon’s choice | N=377  (235 de novo, | Follow-up 104 weeks | 24-month survival 52% for programmable and 50% non-programmable in de novo cohort | Safety and efficacy comparable between valve types; underpowered to assess efficacy within hydrocephalus aetiological subgroups |
| Riva-Cambrin et al, 2016, | Prospective | Manufacturer and programmable vs fixed and shunt failure | N=1036  (Mixed population, 56% under 6 months of age, mean age 131 weeks) | Median follow-up: 264 days (IQR: 68-691) | No difference between programmable vs fixed in shunt failures (HR programmable 0.8 (0.6-1.1) on univariate analysis. Manufacturer or programmable not significant on multivariate analysis (HR 1.0) | No difference between programmable vs fixed types and shunt failure. |
| Kahilogullari et al,  2016 | Retrospective | Fixed pressure vs anti-siphon vs flow control vs adjustable valves. | N=157  (neonates) | Minimum 12 month for study inclusion | No difference in shunt complications among valve types, ultra-small valves had significantly lower complication rates (no p value stated).  [Percentage failure rates not specified] | Rate of early complications lower in ultra-small valves, no difference in valve types. |
| **Single centre** | | | | | | |
| Sinha et al, 2012 | Randomised control trial | Assess Low pressure valves (<5cm) vs medium (5-10cm) | N=40  (82.5% infants (mean age 5 months group A and 3 months group B)) | Mean follow-up 23 months (range: 12-42) | Failure rates not specifically reported, but no difference in shunt revision and complication rates between two groups (p=0.5614) | No difference in low vs medium pressure valves for revision rates and complications. |
| Weinzierl et al, 2008 | Prospective  Single valve | Adjustable differential pressure valve efficacy in preventing overdrainage (Codman-Hakim) | N=15  (<6 months old) | 24 months | Failure rates not reported; valve adjustments not effective in controlling ventricular drainage. | Need randomised studies to elucidate optimal shunt valve designs. |
| Korinth and Gilsbach, 2002 | Prospective  Single valve  Different pressures | Ideal valve pressure in programmable Hakim valves to prevent overdrainage | N=20  (<5 weeks old) | Mean 30.8 months (range: 2-45) | Lower pressure of valve to start with (30-40mmhg) then readjustments over time efficacious. | Hakim programmable set to very low pressure followed by higher in neonates can be used to prevent overdrainage. |
| Martinez-Lage et al, 2007 | Prospective | Assess safety of two programmable valves (Sophy and Polaris) in neonates or <2 months | N=100  (Neonates and infants <2 months of age) | Mean 55 months (+/- 31.1) | 70% had no complications with hydrocephalus controlled; safety and performance similar to other valves. | Programmable valves safe in neonates and infants <2 months old, both types have similar performance to normal valves. |
| Jain et al, 2000 | Prospective | Differential pressure vs flow regulating valves | N=50  (50 infants  72% less than 1-year-old) | Mean 53.8 months (range: 32-70) | Mean shunt life 37.1 months, no difference between types (p=0.72)  Delta flow regulating valves had lower incidence of overdrainage (1 vs 4 cases) | No difference between differential pressure and flow control valves for shunt survival, higher rate of over drainage for differential pressure valves. |
| Henderson et al, 2020 | Retrospective | Adjustable valve (Mitehke ProGav) vs flow-regulated (Orbis-Sigma) | N=55  (infants) | Median 50.4 months | 1-year shunt survival 34%, no difference depending on valve implanted (p=0.18)  Adjustable more likely to cause overdrainage (p=0.02). | Adjustable vs flow regulated valve has no difference in shunt survival, but adjustable more likely to cause overdrainage. |
| Robinson, Kaufman and Park, 2002 | Retrospective | Assess no/low pressure vs medium and high-pressure valves for complications | N=158  86.5% infants (123 vs 23) | Mean 39.8 months (range: 6-99) | 1 year failure rate: 58% in no valve/low-pressure valves vs 31% in medium or high (p=0.0005), with higher number of revisions and overdrainage in low pressure. | Low pressure valves have more complications and higher revision rates. |
| Reed et al, 2020 | Retrospective | Ultrasmall valve vs standard valve | N=156  (<1-year-old, 48 ultra-small valves, 108 regular valves) | Median not reported | No difference in 1-year shunt survival rates (no p value reported), or complications (p=0.56 and 0.23) | No difference in outcomes for ultra-small vs regular valves in infants. |
| Notarianni et al, 2009 | Retrospective | Programmable vs pressure-controlled vs not specified | N=253  (Median age at shunt 30 days old) | Median: 4018 days (range: 1–11,323) | No difference in failure rates between types (p=0.11), number of revisions (p=0.06), and 5-year survival (p=0.14) | No difference between Programmable vs pressure-controlled types and failure rates or 5-year survival. |
| Beez et al, 2013 | Retrospective  Single valve | Fixed pressure paediGAV vs programmable (Codman-Hakim) valve | N=73  (44 GAV, 29 Codman-Hakim  36 <12 months  14 13-60 months  15 >60 months) | Mean: 34 months (range: 0-89) | No difference in shunt failure rates for each valve type (53 vs 56%), no p value reported | No difference between Fixed pressure and programmable types and shunt failure rates. |
| Ahn et al, 2007 | Retrospective  Single valve | Strata programmable valve safety and efficacy | N=53  (Median age 2 yrs) | Mean: 219 days (range: 1-517) | 1-year shunt survival 67.2%, 60.4% adjustment rate | Strata programmable valve is safe and acceptable in infant populations. |
| Gebert et al, 2016, | Retrospective  Single valve | ProGAV valve survival and success rates | N=93  (<12 months old) | Mean: 54.2 months (range: 26-85) | 1-year shunt survival 69.2%, 34.1% at 85 months | ProGAV 1 is safe and reasonable in younger populations who have higher complication rates. |
| Beuriat et al, 2017 | Retrospective | Comparison between Differential pressure (DP) vs Orbis-Sigma Valve (OSV) | N=695  (Median age at shunt 195 days) | Mean: 11 years (+/- 7.4) | DP shunts had significantly worse shunt survival than OSV (HR 1.77, 95% CI 1.19-2.64). | OSV has improved shunt survival over DP valves. |
| Breimer et al, 2011 | Retrospective | Efficacy of ow pressure valves | N=100  (<2 years old) | Mean: 7 years (range not specified) | 1-year shunt survival 42%, 9% overdrainage rate | Use of low-pressure valves is effective in infants, with less high rates of overdrainage reported than expected. |
| Korinth et al, 2003 | Retrospective  Single valve | Programmable hakim valve efficacy | N=40  (<1-year-old) | Mean: 2.6 years (range 2-65 months) | 27.5% compilation rate, 72.5% readjustment rate due to over or under drainage | Programmable shunts reduce infection rates. |
